# Prevalence of Autosomal Monosomy and Trisomy Estimated Using Single Nucleotide Polymorphism Genotype Intensity Chip Information in a Large Population of Juvenile Dairy and Beef Cattle

**DOI:** 10.1111/jbg.12902

**Published:** 2024-10-14

**Authors:** Cliona A. Ryan, Deirdre C. Purfield, Daragh Matthews, Claudia Rathje, Ainhoa Valldecabres, Donagh P. Berry

**Affiliations:** ^1^ Department of Animal Bioscience Teagasc, Moorepark Co. Cork Ireland; ^2^ Department of Biological Science Munster Technological University Co. Cork Ireland; ^3^ Genetics Department Irish Cattle Breeding Federation Co. Cork Ireland; ^4^ School of Biosciences University of Kent Canterbury UK

## Abstract

Aneuploidy, a genetic condition characterised by the deletion (monosomy) or duplication (trisomy) of a chromosome, has been extensively studied in humans, particularly in the context of trisomy on chromosome 21, also known as Down syndrome. Research on autosomal aneuploidy in live‐born cattle has been limited to case reports, resulting in a lack of prevalence estimates of aneuploidy in cattle. Furthermore, the viability or lethality of aneuploidy on specific autosomes in cattle has not been well documented. The objective of this study was to estimate the prevalence of autosomal aneuploidy in a large population of new‐born and juvenile beef and dairy cattle using single nucleotide polymorphism (SNP) chip genotype intensity data. Of the population of 779,138 cattle genotyped when younger than 15 months of age, 139 cattle (i.e., 0.017%) were diagnosed with one case of autosomal trisomy. Trisomy in only 10 different autosomes were detected (BTA 4, 6, 12, 15, 20, 24, 26, 27, 28 and 29) albeit the one case of trisomy detected on *Bos taurus* autosome (BTA) 4 was in an additional population of 341,927 cattle that were genotyped at > 15 months of age and was therefore excluded from prevalence estimates to minimise bias. The prevalence of trisomy per chromosome was generally inversely related to the length of the chromosome. Although the number of affected individuals was few, there was no evidence of differences in prevalence by breed, inbreeding level or parental age. The parental origin of the detected cases of trisomy was maternal for 92% of the cases. No cases of monosomy were detected despite the large dataset, which included calves genotyped at birth, indicating the potential lethal nature of monosomy in cattle. Cytogenetic testing was used to verify three of the animals with detected autosomal trisomy who were still alive. Eighteen of the 139 animals identified with autosomal trisomy were recorded as being stillborn, resulting in a prevalence of autosomal aneuploidy in live‐born cattle of 0.015%. Of the 121 live‐born cattle with autosomal trisomy, a total of 68 died on farm at, on average (standard deviation), 6.8 (8.7) months of age. All animals with autosomal trisomy on BTA 6, 12, 15, 20 or 24 were either stillborn or died on farm within 15 days of birth. This study is the first report of trisomy on BTA 4, 6, 15, 20 and 27 in live‐born cattle, as well as the first to document fertile cows with trisomy on BTA 4, 27 or 28. Given that genotype intensity SNP data from SNP‐chips are readily available, identifying animals affected with autosomal aneuploidy as well as quantifying and monitoring the incidence can be easily undertaken.

## 1 Introduction

Many chromosomal structural rearrangements or numerical aberrations have been reported in cattle (Ducos et al. 2008; Berry et al. 2017; Ryan et al. 2024). The majority of these have been associated with infertility, embryonic or fetal loss, anatomical defects or severe congenital malformations (Raudsepp and Chowdhary 2016; Iannuzzi, Parma, and Iannuzzi 2021). Such impact has obvious monetary implications for breeders and producers alike. One type of chromosomal numerical aberration is aneuploidy, which is a genetic condition characterised by the deletion (i.e., monosomy) or duplication (i.e., trisomy) of a chromosome (Hassold and Hunt 2001). Aneuploidy has been reported to be present in at least 30% of bovine oocytes (Nicodemo et al. 2010), with sex‐chromosome aneuploidy often resulting in infertility (Abir et al. 2001; Iannuzzi, Parma, and Iannuzzi 2021), while autosomal aneuploidy predominantly leads to abortions (Coates, Schmutz, and Rousseaux 1988).

Trisomy has been separately detected on every autosome causing spontaneous abortions in humans, with the prevalence varying by autosome (Kuhn et al. 1987; Dunn, Grunfeld, and Kardon 2001). Trisomy has also been reported on every autosome other than 1 and 11 in live‐born humans, but trisomy in autosomes other than 13, 18 and 21 are rare (Gersen and Keagle 2013). Autosomal monosomy, on the other hand, is extremely rare in both living individuals and aborted foetuses (Gersen and Keagle 2013). Autosomal aneuploidy is also a rare phenomenon in live‐born cattle, with the majority of cases being reported to be culled by breeders due to severe congenital malformations (King 2008). For example, a Hereford calf with trisomy on chromosome 28 was reported to have slow growth rate, brachygnathia, hypersalivation, strabismus convergence and two cervixes, as well as other anatomical defects (Iannuzzi et al. 2001). Previous studies have reported autosomal trisomy on chromosomes 12, 16, 18 and 22 to also be associated with lethal brachygnathia in cattle (Herzog, Höhn, and Rieck 1977; Herzog and Hoehn 1991; Agerholm and Christensen 1993).

Given that the majority of the autosomal aneuploidy studies in cattle have been case reports, estimates of the prevalence of aneuploidy per autosome do not exist for cattle. The objective of this study was to estimate the prevalence of autosomal aneuploidy in a large population of juvenile beef and dairy cattle using readily available single nucleotide polymorphism (SNP) chip genotype intensity data and to investigate whether the prevalence of aneuploidy was influenced by breed, parental age, level of inbreeding as well as the length of the autosome on which aneuploidy occurred. An additional objective was to further develop the methodology proposed by Ryan et al. (2024) for aneuploidy detection on sex chromosomes and integrate a screening tool for autosomal aneuploidy into existing SNP chip genotype pipelines used for parentage verification, sex prediction and genomic evaluations.

## 2 Materials and Methods

### 2.1 Genotype Data

SNP genotype intensity data from the International Dairy and Beef Version V5 chip based on the custom Thermo Fisher Scientific genotype platform were available for 779,138 dairy and beef cattle, consisting of 574,749 producer‐recorded females and 204,389 males. Of these, 225,781 animals were purebred dairy or beef, and the remaining 553,357 were crossbred cattle. Of the purebreds, 51% were Holstein‐Friesian, while 49% were beef breeds (96% were either Angus, Aubrac, Charolais, Hereford, Limousin, Salers, Shorthorns or Simmental). All animals were under the age of 15 months at the time of genotyping and had a call rate of ≥ 90%. The custom genotype panel consists of 49,147 SNPs on the UMD 3.1 build. Only autosomal SNPs with a locus call rate ≥ 90% were retained. Following edits, 48,502 SNPs remained. The overall incidence of autosomal aneuploidy in the present study was based on the 779,138 animals genotyped before reaching 15 months of age, which is the age at which dairy and beef females are served for calving at 24 months of age in seasonal calving systems as exist in Ireland (Berry et al. 2013); hence, this incidence rate should have minimal bias due to infertility. SNP genotype intensity data were also available for an additional 341,927 beef and dairy animals (287,142 females and 54,785 males) that were genotyped ≥ 15 months of age. These animals were also screened for aneuploidy, and any cases detected are included in the description later discussed of animals detected with autosomal trisomy; however, they were not included in the calculation of the prevalence of aneuploidy in order to minimise bias.

### 2.2 Genotype Intensity Data

Possible cases of autosomal monosomy or trisomy were detected in the present study using the principles proposed by Ryan et al. (2024) to detect sex‐chromosome aneuploidy based on the BAF, LRR, and *R* values using Illumina (Illumina Inc.) SNP genotypes; this approach was modified to detect autosomal aneuploidy using Thermoscientific (Thermo Fisher Scientific Inc.) SNP genotypes. The *R* value represents the sum of the raw signal intensity channels for each of the fluorescent dyes associated with the A and B alleles called for each SNP. The LRR is log_2_ of the observed *R* value divided by the expected *R* value relative to a reference sample (Peiffer et al. 2006). An LRR value of zero indicates a neutral copy number, a positive LRR value indicates copy number gain and a negative LRR value indicates copy number loss (Hashem et al. 2016). The BAF is an estimate of N_B_/(N_A_+N_B_) based on the normalised intensity of both alleles, where N_A_ and N_B_ are the number of A and B alleles called per SNP, respectively (Staaf et al. 2008).

Alleles are assigned a value of A or B in the reported genotype file. Therefore, a set of consecutive SNPs for one animal will appear in a diploid BAF whole‐genome Manhattan‐type plot as horizontal bands at 0 (AA), 0.5 (AB) or 1 (BB). In the presence of chromosomal duplication, heterozygous BAF bands typically appear around the values of 0.33 (AAB) and/or 0.67 (ABB), between the homozygous BAF bands at 0 (AAA) and 1 (BBB). The LRR and *R* values for a duplication will be greater than their respective values on a normal diploid chromosome. Conversely, in the case of a chromosomal deletion, the only two possible BAF bands occur at 0 (A) and 1 (B), along with lower LRR and *R* values.

### 2.3 Detecting Aneuploidy

For each animal, the mean LRR and the mean *R* value of all SNPs with a call rate ≥ 90% on the autosome being investigated were calculated. These values were expressed in standard deviation units relative to the average and standard deviation of the LRR and *R* values of SNPs across all autosomes (excluding the autosome being investigated) for that particular individual. The terms ‘standardised LRR’ and ‘standardised *R* values’ will be used henceforth to refer to these calculated statistics. For each autosome, the population mean and the standard deviation of the standardised LRR and *R* values were calculated. Additionally, for each animal, the percentage of SNPs on the autosome under investigation that had a BAF in the expected heterozygous range of 0.45–0.55 for a diploid genome was calculated.

For each autosome, animals with ≤ 1% of the SNPs for the autosome under investigation in the BAF range of 0.45–0.55 (inclusive) were identified. Subsequently, those animals meeting this criterion and having both the standardised LRR and standardised *R* values that deviated more than ±3 standard deviations from the respective mean standardised values in the population for the autosome under investigation were classified as having autosomal aneuploidy.

Animals classified with monosomy for the autosome under investigation would exhibit standardised genotype intensity values that were < −3 standard deviations from the respective mean standardised values in the population. Animals classified with trisomy for the autosome under investigation would display standardised genotype intensity values that were > 3 standard deviations from the respective mean standardised values in the population. To visually explore and confirm detected cases of possible autosomal aneuploidy, the LRR and BAF for suspect animals were visualised using Manhattan plots for the entire genome.

Where possible, animals classified as having aneuploidy were parentage verified. Where both parental genotypes were available for the animal diagnosed with aneuploidy, SNPs on the autosome under analysis with opposing parental homozygotes were compared to the genotype of the animal with autosomal aneuploidy, enabling the determination of which parent contributed the extra allele in the case of trisomy or the sole allele in the case of monosomy at that specific genomic position. Where possible, the age of the parental contributor of aneuploidy at the time of the birth of the progeny was investigated; sire age was only investigated for animals born from natural mating as the age of the sire when the ejaculate for artificial insemination was collected was not available. If the animal with aneuploidy had genotyped progeny, the progeny also underwent parentage verification to ensure that the animal with autosomal aneuploidy truly was fertile and that the assigned progeny were not due to a parentage recording error.

### 2.4 Inbreeding

Genomic inbreeding coefficients were calculated for all animals with aneuploidy based on the autosomal runs of homozygosity. The autosome affected by aneuploidy in each animal was excluded from the inbreeding calculation.

### 2.5 Cytogenetic Analysis

Cytogenetic analysis was conducted at the University of Kent on one female exhibiting trisomy on Bos Taurus Autosome (BTA) 26 and two animals with trisomy on BTA 27. No animals with monosomy were detected in the present study. Blood samples were obtained from the coccygeal vessels using 10 mL lithium heparin evacuated tubes (BD Vacutainer, LH 102 I.U.; BD, Plymouth, UK). To prepare the blood samples for karyotype analysis, heparinised blood was cultured in PB MAX Karyotyping medium (Gibco, Grand Island, NY) at 37°C and 5% CO_2_ for 96 h. Cell division was arrested by the addition of colcemid (Gibco) at a concentration of 10.0 μg/mL for 30 min, followed by hypotonic treatment using 75 mM potassium chloride. A mixture of methanol and acetic acid in a 3:1 ratio was added on top of the hypotonic solution and the tubes were inverted for flash fixation. Metaphases for karyotyping were stained with 4′,6‐diamidino‐2‐phenylindole in VECTASHIELD antifade medium (Vector Laboratories, Burlingname, CA). Image capturing was conducted using an Olympus BX61 epifluorescence microscope equipped with a cooled charge‐coupled device camera and the SmartCapture software (Digital Scientific, Cambridge, UK) for a total of 20 metaphases per sample. Karyotyping was performed on at least 10 of the 20 captured metaphases per sample with the assistance of SmartType software (Digital Scientific), and the chromosomes were arranged following the International System for Chromosome Nomenclature of Domestic Bovids (Cribiu et al. 2001).

## 3 Results

### 3.1 Prevalence

Of the juvenile population of 779,138 cattle genotyped when younger than 15 months of age, 139 cattle (i.e., 0.017%), including 89 females and 49 males, were diagnosed with autosomal trisomy; trisomy in 9 different autosomes were detected (BTA 6, 12, 15, 20, 24, 26, 27, 28 and 29; Table 1). The prevalence in the purebred and crossbred populations was 0.01% and 0.02%, respectively. Of the purebred dairy animals, 0.001% were diagnosed with trisomy, while 0.01% of the purebred beef animals were diagnosed with trisomy (Table 2). No animal exhibited trisomy on more than one autosome. Trisomy was most commonly detected on the shorter autosomes, particularly BTA 27, which accounted for half (i.e., 69) of the identified cases of trisomy in the sample population. No case of autosomal monosomy was detected.

**TABLE 1:** The number of live‐born and stillborn animals identified with autosomal trisomy in the juvenile population of 779,138 cattle genotyped < 15 months of age and number of animals identified with autosomal trisomy in the population of 341,927 animals genotyped ≥ 15 months of age.

Autosome	Live‐born and stillborn cattle with trisomy genotyped at < 15 months of age	Live‐born cattle with trisomy genotyped at < 15 months of age	Live‐born cattle with trisomy genotyped at ≥ 15 months of age	Number of animals with progeny genotyped at any stage of life
4	0	0	1	1
6	1	1	0	0
12	5	1	0	0
15	4	3	0	0
20	7	6	0	0
24	2	2	0	0
26	14	13	3	0
27	69	68	6	4
28	35	28	1	1
29	2	1	0	0
Total	139	123	11	6

**TABLE 2:** The number of animals with trisomy genotyped at different stages of life, the autosomes trisomy occurred on and the number of purebred and crossbred animals with autosomal trisomy.

Age at genotyping and progeny status	Number of animals with trisomy	Autosome with trisomy (number of affected animals)	Number of purebred animals	Breed of purebreds (number of affected animals)^a^	Number of crossbred animals
≤ 15 days and had no progeny	40	12 (1), 15 (4), 20 (3), 24 (1), 26 (6), 27 (11), 28 (13), 29 (1)	6	CH (1), HO (2), LM (1), UN (2)	31
Between 15 days and 15 months and had no progeny	97	6 (1), 12 (4), 20 (4), 24 (1), 26 (8), 27 (56), 28 (22), 29 (1)	17	AA (5), CH (2), HE (1), HO (3), IM (1), LM (4), SI (1)	80
≥ 15 months and had no progeny	7	26 (3), 27 (4)	2	LM (2)	5
Had progeny	6	4 (1), 27 (4), 28 (1)	3	HE (1), LM (1),SH (1)	3

^a^ Angus (AA), Charolais (CH), Hereford (HE), Holstein (HO), Irish Moile (IM), Limousin (LM), Shorthorn (SH), Simmental (SI), and Unknown (UN).

**FIGURE 1:**
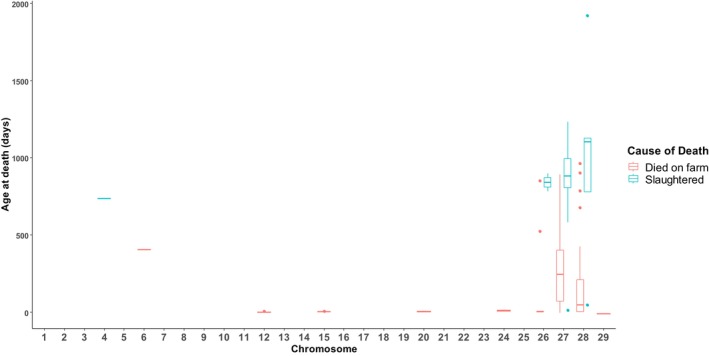
Box and whisker plot of the age of death, in days, for all animals with trisomy on chromosome 4, 6, 12, 15, 20, 24, 26, 27, 28 or 29 in the population of 1,121,065 animals genotyped at any stage of life that either died on farm or were slaughtered in an abattoir. The box represents the interquartile range of the age at death for each chromosome, and the line inside the box represents the median. The whiskers extend from the edges of the box to reflect the range of the data, excluding outliers, and the points represent the outliers. [Colour figure can be viewed at wileyonlinelibrary.com]

**FIGURE 2:**
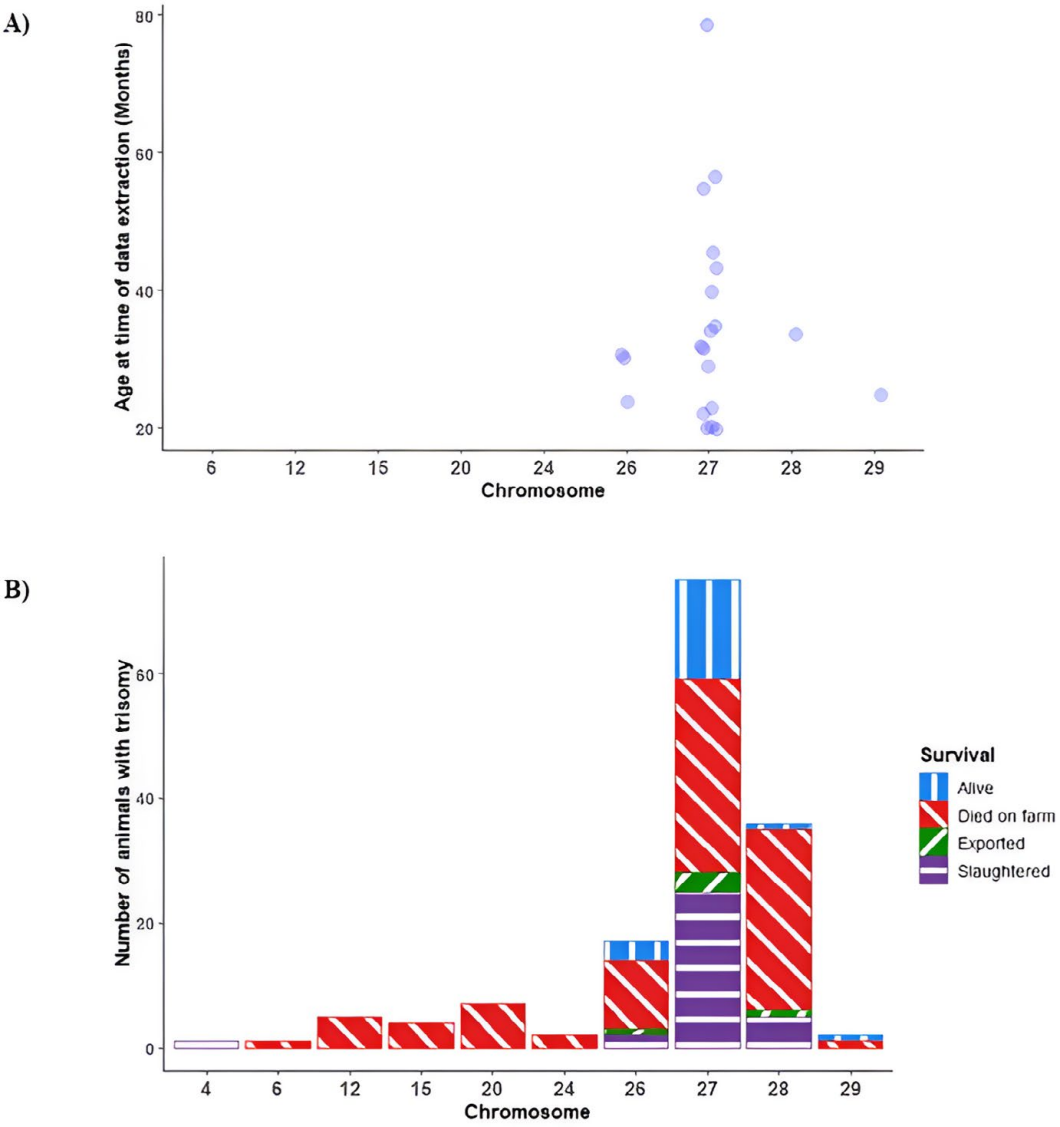
(A) Scatterplot of the age of animals, in months, for all animals with autosomal trisomy in the population of 1,121,065 animals genotyped at any stage of life that were still alive at the time of data extraction. (B) The reason recorded for an animal no longer existing at the time of data extraction (including those still alive) for each animal identified with trisomy on chromosome 4, 6, 12, 15, 20, 24, 26, 27, 28 or 29 in the population of 1,121,065 animals genotyped at any stage of life. [Colour figure can be viewed at wileyonlinelibrary.com]

Of the 139 animals identified with autosomal trisomy, 42 were genotyped younger than 15 days old (Table 2), including 18 that were recorded as stillborn. The remaining 97 animals were genotyped between 15 days and 15 months of age, leading to a prevalence of autosomal aneuploidy in live‐born cattle of 0.015% (Table 1). Of the 121 live‐born cattle with autosomal trisomy, 68 died on the farm at, on average, 6.8 months (standard deviation 8.7 months) of age. All 19 animals with autosomal trisomy on BTA 6, 12, 15, 20 and 24 died on farm within 15 days of life (Figure 1). Of the remaining 144 animals with autosomal aneuploidy, 91 died on farm (Figure 1), 31 were slaughtered in the abattoir, five were exported and 21 were still alive at the time of data extraction, with the ages of the animals that were still alive at the time of data extraction ranging from 19 to 78 months (Figure 2).

The calculated mean inbreeding coefficient was 0.03 for the animals diagnosed with trisomy, excluding the autosome with trisomy in the inbreeding calculation. The mean inbreeding coefficient in the overall population of cattle was 0.04.

An additional 11 cases of autosomal trisomy (10 females and 1 male) were detected in the population of 341,927 animals that were genotyped when older than 15 months of age—one on BTA 4, three on BTA 26, six on BTA 27 and one on BTA 28 (Table 1). In the whole population of 1,121,065 animals genotyped at any stage of life, only six animals (two of which were genotyped younger than 15 months of age) of the 150 diagnosed with autosomal aneuploidy had progeny.

The whole‐genome BAF and LRR Manhattan plots of one exemplar animal from each autosome with detected autosomal aneuploidy are shown in Figure S1. For each animal, the BAF plot revealed four clusters around the values of 0 (AAA), 0.33 (AAB genotype), 0.67 (ABB genotype) and 1 (BBB genotype) for the specific autosome with a duplication, whereas the other diploid autosomes had three clear clusters of BAF at values of 0 (AA), 0.5 (AB) and 1 (BB). On the autosome with a duplication, the LRR values per SNP were higher than the remaining diploid autosomes.

**FIGURE 3:**
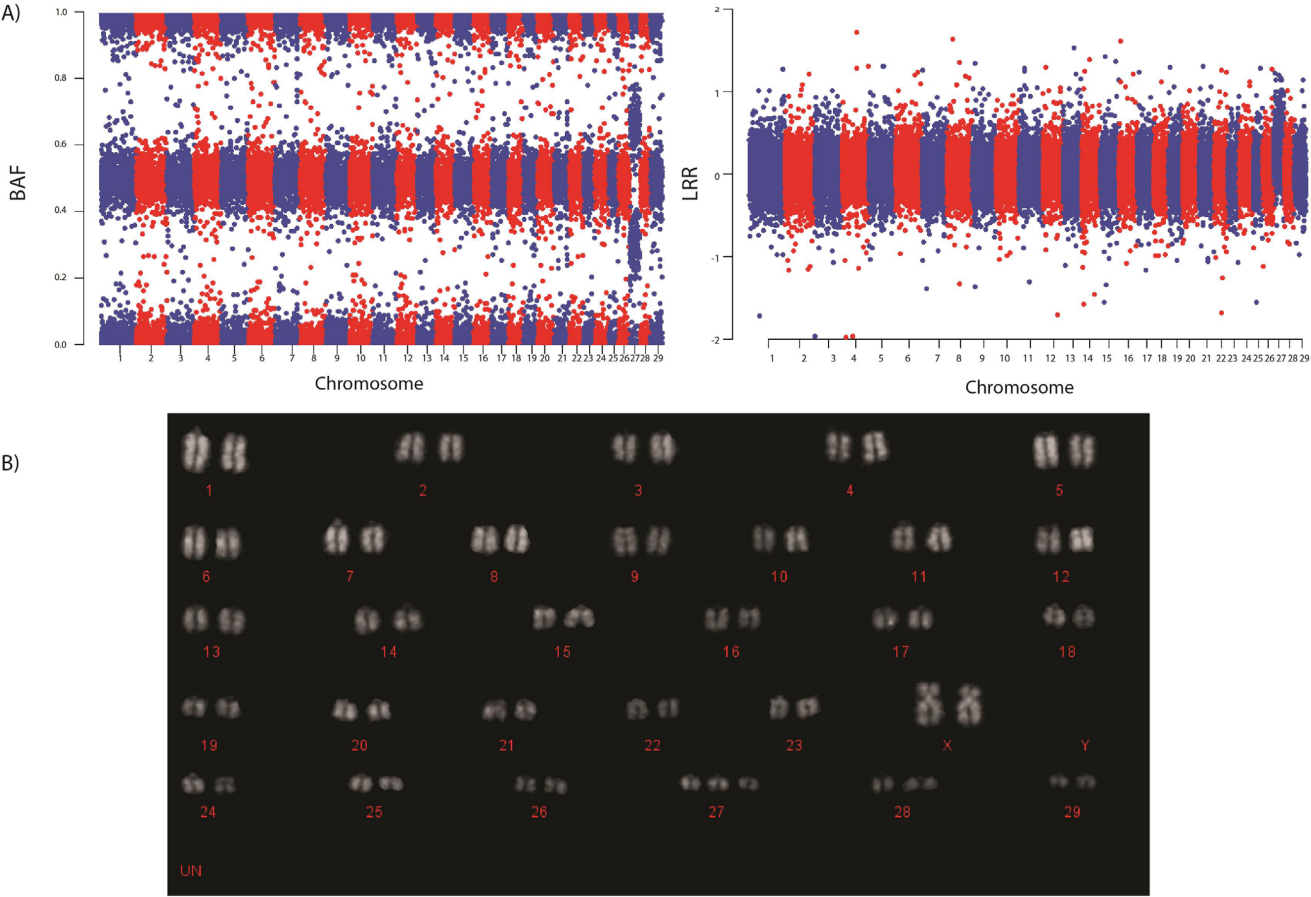
(A) Real life examples of log *R* ratio (LRR) and B‐allele frequency (BAF) plots for an animal with trisomy on chromosome 27. (B) Karyotype of a heifer carrying a trisomy on chromosome 27. [Colour figure can be viewed at wileyonlinelibrary.com]

Two of the females with trisomy on BTA 27 and one female with trisomy on BTA 26 were still alive at the time of analysis and were all confirmed by karyotype analysis to have autosomal trisomy (Figure 3). Given the fact that at least 10 metaphases were examined by karyotyping and all demonstrated autosomal trisomy, mosaicism of ≥ 3% at the autosome with trisomy can be excluded for both with 99% confidence, or mosaicism of ≥ 2% can be excluded with 95% confidence (Hook 1977).

### 3.2 Trisomy on Long Autosomes: BTA 4 and 6

One animal with trisomy on BTA 4 and one animal with trisomy on BTA 6 were identified in the whole population of the 1,121,065 animals that were genotyped at any life stage. The animal with trisomy on BTA 4 was a fertile Jersey‐Holstein cross female slaughtered at 24 months of age after giving birth to a stillborn bull calf that was not genotyped. The animal with trisomy on BTA 6 was a purebred Hereford male that died on farm at 13 months of age.

### 3.3 Lethal Trisomy on BTA 12, 15, 20 and 24

All 17 animals in the whole population of 1,121,065 animals that were diagnosed with trisomy on BTA 12, 15, 20 or 24 died within 15 days of being born (Figure 1). Specifically, of the five calves with trisomy on BTA 12, four were stillborn, and the remaining calf died within 5 days of birth. Similarly, all four calves with trisomy on BTA 15 died within 5 days of birth, including one calf recorded as being stillborn. All seven calves with trisomy on BTA 20 died before reaching 8 days of age, while the two calves with trisomy on BTA 24 died at five and 15 days of age. Therefore, based on the results from the present study, trisomy on BTA 12, 15, 20 or 24 is likely lethal (Figure 1).

### 3.4 Trisomy on Short Autosomes: BTA 26, 27, 28 and 29

While a large proportion of animals with trisomy on BTA 26, 27, 28 and 29 identified in the whole population of 1,121,065 animals genotyped at any stage of life were stillborn or died on farm (Figure 2), other animals with trisomy on these autosomes were still alive at the time of data extraction. The ages of the animals with trisomy on BTA 26, 27, 28 or 29 that were still alive at the time of data extraction ranged from 19 to 78 months (Figure 2), indicating variability in the degree of lethality for trisomy on these autosomes. For instance, of the 17 animals with trisomy on BTA 26, nine (64%) died on the farm while an additional calf was stillborn. Of those nine that died on the farm, seven were younger than 7 days old at the time of death, while the other two died at 17 and 28 months of age (Figure 1). Of the remaining seven animals with trisomy on BTA 26, one was exported, two were slaughtered in the abattoir (Figure 2) and four were still alive at the time of analysis, with ages ranging from 23 to 64 months (Figure 2).

Similarly, of the 75 animals with trisomy on BTA 27, 30 (40%) died on the farm, and an additional calf was stillborn. Excluding the stillborn calf, the age of death of the other 30 animals that died on the farm ranged from 6 days of age to 30 months of age, with the mean (standard deviation) age of death being 8.9 months (7.9 months). Despite the high mortality rate for animals with trisomy on BTA 27, four females with trisomy on BTA 27 (5.3% of the females with trisomy on BTA 27) were fertile and had progeny; one female had three progeny, another female had two progeny, and the remaining two females had one progeny each. None of the progeny of females with trisomy on BTA 27 were genotyped. Of the remaining 44 animals with trisomy on BTA 27, 25 were slaughtered in the abattoir, three were exported and 16 were still alive at the time of analysis (Figure 2), with ages ranging from 19 to 78 months at the time of data extraction (Figure 2).

Of the 36 animals with trisomy on BTA 28, 22 (61%) died on the farm, with an additional seven being stillborn. Excluding the stillborn calves, the age of death of the other 22 animals that died on farm ranged from 2 days to 32 months, with the mean (standard deviation) age of death being 6.1 months (9.4 months). However, at least one female with trisomy on BTA 28 was fertile and had two progeny, highlighting the potential for survival and reproductive success despite the high mortality rate associated with the trisomy. Of these two progeny, one was genotyped and parent verified and had a normal karyotype based on the available SNP data. This female that had two progeny was the only animal with trisomy on BTA 28 that was still alive at the time of analysis, and she was 33 months of age at the time of data extraction. Of the remaining six animals with trisomy on BTA 28, five were slaughtered in the abattoir, and one was exported (Figure 2).

Only two animals with trisomy on BTA 29 were identified, one of which was alive at the time of data extraction (25 months of age), while the other was stillborn.

### 3.5 Parental Origin

Parental genotypes were available for 134 of the 150 animals that were diagnosed with autosomal trisomy in the whole population of 1,121,065 animals genotyped at any stage of life; of these, 124 (92%) inherited the extra chromosome from the dam. The mean age of the 124 dams confirmed to be the parental origin of aneuploidy when they gave birth to the animal with autosomal trisomy varied from 22 months to 17 years, with a mean (standard deviation) calving age of 5.8 years (3.7 years). In comparison, the mean (standard deviation) age of all the dams for the entire population of 1,121,065 animals when they gave birth was 5.2 years of age (2.4 years). Of the 124 dams, 28 were primiparous. The 124 dams had 610 other progeny (excluding the progeny identified with trisomy), 320 of which were genotyped and, of these, all had normal karyotypes. The mean calving interval of these dams was 371 days.

Of the 10 animals that inherited the extra chromosome paternally, six of the 10 sires were artificial insemination (AI) bulls. The age of the AI bulls at semen collection for the service leading to the birth of the calf was not available. The age of the four natural mating bulls was 4.2, 5.1, 8.8 and 10.5 years when the affected progeny were born, with a mean (standard deviation) age of 7.1 years (2.9 years). In comparison, the mean age (standard deviation) of the natural mating bulls in the entire population of 1,121,065 animals when their progeny was born was 5.3 years (2.6 years). The four natural mating bulls each had 150, 132, 70 and 59 other progeny (excluding the progeny identified with trisomy), 306 of which were genotyped and all of which had normal karyotypes.

## 4 Discussion

The majority of autosomal aneuploidy studies in live‐born cattle have consisted of case reports, lacking comprehensive population‐based autosomal aneuploidy screening analysis. Case reports have only documented trisomy on certain autosomes, which are likely those autosomes where animals with trisomy materialises in phenotypic malformations, thereby distinguishing these animals as candidates for a case study. The study herein discovered that some females with autosomal aneuploidy are fertile and, in fact, had multiple progeny; therefore, it is likely that these females do not have any phenotypic abnormalities and therefore would never be chosen for a case study as they likely remain undetected in the herd. Consequently, the limited scope of case reports has resulted in the failure to detect trisomy on certain autosomes, such as BTA 4, 6, 26 and 27, all of which were detected in the present study but not reported previously. The extensive dataset of 779,138 animals used in the present study provides a high level of confidence in the estimated prevalence of all viable autosomal trisomy cases in cattle.

Prior investigations have not identified cases of monosomy in live‐born cattle, potentially attributed to small sample sizes or the death of affected animals shortly after birth, thereby hindering the detection of monosomy. Of note in the present study was the absence of any detected monosomy cases despite the comprehensive dataset, which included calves sampled at birth and stillborns, further indicating the potential lethal nature of monosomy in cattle.

### 4.1 Prevalence of Aneuploidy

Aneuploidy has been reported to be present in at least 30% of bovine oocytes (Nicodemo et al. 2010), and bovine embryonic aneuploidy has been detected on all autosomes, except for BTA 7 (Silvestri et al. 2021). However, most of these cases of embryonic aneuploidy have not been observed in live‐born cattle due to their expected lethality, resulting in abortions at various stages of gestation (Mayr et al. 1985; King 2008; Häfliger, Seefried, and Drögemüller 2020). Therefore, it is likely that the true prevalence of aneuploidy cases are underestimated in the present study since no data were available on aborted embryos or foetuses.

While it is well documented from human and animal studies that aneuploidy can occur on any chromosome (Hassold, Hall, and Hunt 2007; Silvestri et al. 2021), it appears that in cattle, the viability of embryos with autosomal aneuploidy is dependent on the specific autosomes that have aneuploidy. A similar phenomenon is observed in humans, whereby trisomy on autosomes other than 13, 18 and 21 are rare in live‐born individuals (Gersen and Keagle 2013). Synteny regions between Homo sapiens (HSA) 13 and BTA 12, HSA 18 and BTA 24 and HSA 21 and BTA 1 have been reported (Sun et al. 1997; Band et al. 2000; Drögemüller et al. 2005). The lethality of embryonic aneuploidy has been attributed to the imbalance effects of several major genes together with many minor genes specific to the individual chromosome (Holečková et al. 2021).

It has been suggested that the incidence of aneuploidy is inversely related to the length of the chromosome (Torres, Williams, and Amon 2008), which is consistent with the findings from the present study. With the exception of one animal with trisomy on BTA 4, the trisomy detected in the present study among animals that survived past 24 months of age was limited to shorter autosomes, specifically BTA 26, 27, 28 or 29, with BTA 27 accounting for over half of the observed cases of trisomy. The majority of previous case reports of autosomal trisomy in cattle (Holečková et al. 2021) and horses (Brito et al. 2008; Lear and Bailey 2008; Bugno‐Poniewierska and Raudsepp 2021) have documented autosomal trisomy cases on the shorter autosomes, likely because shorter autosomes tend to carry fewer genes; therefore their effects are usually not as detrimental as aneuploidy on longer autosomes (Ducos et al. 2000). This is consistent with the findings from the present study, as BTA 27, which had the greatest incidence of trisomy, also has the fewest number of genes of all the autosomes based on the bovine UMD 3.1 reference genome. Additionally, no case of aneuploidy was detected on BTA 3 in the present study or in previous studies, which contains the most genes of all the bovine autosomes.

### 4.2 Lethality

Previous case reports on autosomal aneuploidy have documented non‐mosaic autosomal trisomy in live‐born or stillborn cattle, as well as aborted foetuses, on BTA 12, 17, 18, 20, 21, 22, 24, 27, 28 and 29 (Herzog and Hoehn 1991; Agerholm and Christensen 1993; Lioi, Scarfi, and Di Berardino 1995). However, the literature has only reported that trisomy on BTA 24 and 28, and occasionally BTA 22, are non‐lethal. Traditionally, the classification of trisomy on a particular autosome as lethal has been based on single case studies involving aborted fetuses, stillborn calves or calves with autosomal trisomy that died shortly after birth. Contrary to these previous findings, viable animals with trisomy on BTA 4, 26, 27, 28 and 29 were identified in the present study. For example, the only previous reports of trisomy on BTA 27 and 29 were documented in stillborn calves with severe congenital anomalies (Coates, Schmutz, and Rousseaux 1988; Häfliger, Seefried, and Drögemüller 2020), leading to the previous consideration of trisomy on these autosomes as lethal in bovines. The current study, however, clearly demonstrates that the phenotypic manifestation of trisomy on BTA 27 and 28 can range from a stillborn calf to a fertile cow. In comparison, the absence of any animals with trisomy on BTA 12, 15, 20 or 24 surviving past 15 days of age suggests that trisomy on these autosomes is likely lethal.

The age at death varied for animals with trisomy on BTA 26, 27 and 28 in the current study; 46 of these animals were still alive after 1 month, 16 of which were still alive after 12 months and six of which died on the farm after 24 months of age. This variability in age of death indicates that even if animals with trisomy surpass the critical 15‐day mark, which is when most animals carrying a trisomy died in the current study, they may still have a shorter life expectancy relative to their contemporaries. Consequently, early identification of animals with trisomy is crucial for informed management decisions. This is similar to trisomy cases in humans, where a shorter life expectancy is also observed for individuals with trisomy on chromosome 21 (i.e., Down syndrome). In the 1940's, the average life expectancy of humans with Down syndrome was 12 years (Penrose 1949) and although this has increased to mid‐ to late‐50's due to medical improvements, it is still substantially below that of the general population (Janicki et al. 1999; Glasson et al. 2002).

### 4.3 Parental Origin of Aneuploidy

The occurrence of aneuploidy is primarily attributed to errors during maternal meiosis, with maternal errors predominantly occurring during meiosis I (Hassold and Hunt 2001; Gabriel et al. 2011); the incidence of these errors increases with maternal age in humans (Hassold, Hall, and Hunt 2007). Based on 782 humans with Down syndrome (i.e., trisomy on chromosome 21), the extra chromosome originated from the mother in over 90% of the cases (Hassold, Hall, and Hunt 2007), corroborating the observations from the present study. Similarly, in the single in vitro fertilisation‐produced cattle embryo identified with trisomy on both BTA 14 and 19 reported by Bouwman and Mullaart (2023), the extra chromosome was inherited from the dam. Furthermore Silvestri et al. (2021) reported that 83 of the 113 bovine embryos detected with trisomy across all autosomes other than BTA 7 and 21 were due to errors during maternal meiosis 1.

While the risk of aneuploidy increases with maternal age in humans (Hassold, Hall, and Hunt 2007), the wide range in ages of dams that had progeny with autosomal trisomy in the present study suggests no clear association between age and aneuploidy risk in cattle. Similarly, Silvestri et al. (2021) reported that the age of the donor dam in their in vitro‐produced bovine embryos had no effect on aneuploidy incidence. Furthermore, no association between maternal age and aneuploidy was detected in porcine oocytes (Hornak et al. 2011). Although the mean age of the natural mating bulls confirmed to be the parental origin of aneuploidy at the time when the animal in question was born was 1.8 years older than the mean age of the natural mating sires at the time of the birth of their progeny in the entire population, this was only based on a very small sample size of four sires that were the parental origin of aneuploidy.

## 5 Conclusion

A technique for identifying autosomal aneuploidy using genotype intensity metrics was proposed and applied to 1,121,065 animals; cytogenetic testing was used to validate three females with detected autosomal trisomy. The large dataset used in the present study revealed that trisomy on BTA 4, 26, 27, 28 and 29 can indeed be viable, whereas trisomy on BTA 6, 12, 15, 20 and 24 may always be lethal. Monosomy is likely also lethal, as no cases were detected in the large dataset used in the present study. This study is the first report of trisomy on BTA 4, 6, 15, 26 and 27 in live‐born cattle. Furthermore, this study revealed the first cases of fertile cows with trisomy on BTA 4, 27 and 28. Of the juvenile population of 779,138 cattle genotyped when younger than 15 months of age, 139 cattle (i.e., 0.017%) were diagnosed with autosomal trisomy. While the prevalence is low, the proposed approach described in the present study using often readily accessible SNP chip data can be automated thus providing a screening tool at minimal cost facilitating early decisions about the fate of the affected animals thereby reducing the potential for further monetary losses.

## Conflicts of Interest

“Donagh P. Berry” is an Editorial Board member of Journal of Animal Breeding and Genetics and a co‐author of this article. To minimise bias, he was excluded from all editorial decision‐making related to the acceptance of this article for publication.

## Supporting information


Figure S1.

## Data Availability

The data used in the present study originated from a pre‐existing database managed by the Irish Cattle Breeding Federation (ICBF). Data available on request from the authors.
