# Supplementary material for: Prevalence of Autosomal Monosomy and Trisomy Estimated Using Single Nucleotide Polymorphism Genotype Intensity Chip Information in a Large Population of Juvenile Dairy and Beef Cattle
Source: J Anim Breed Genet. 2024 Oct 14;142(3):277–86. doi: 10.1111/jbg.12902 (PMC11975166; doi:10.1111/jbg.12902)
Supplement: Supplementary file 1 — Figure S1. [file JBG-142-277-s001.docx]

**SUPPLEMENTARY APPENDIX**

| 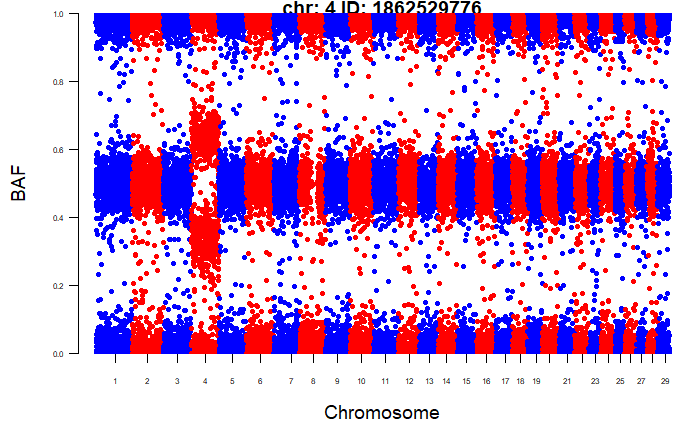A) | 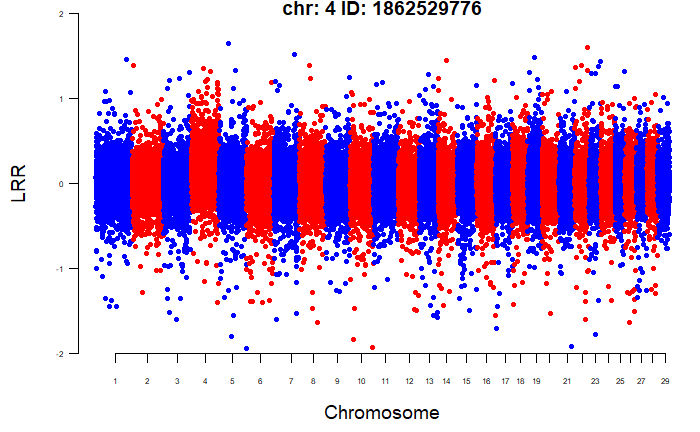 |
| --- | --- |
| 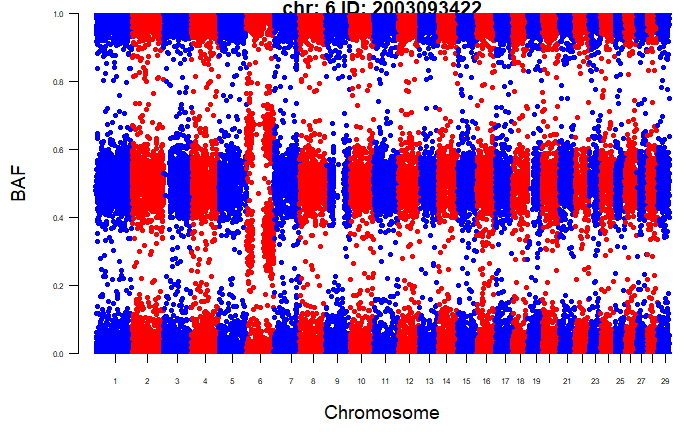B) | 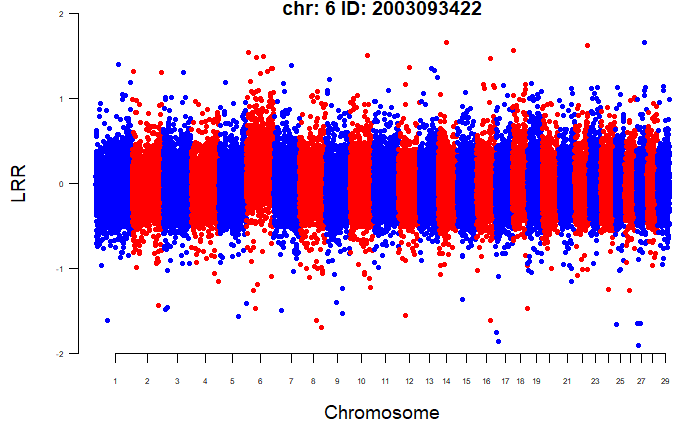 |
| 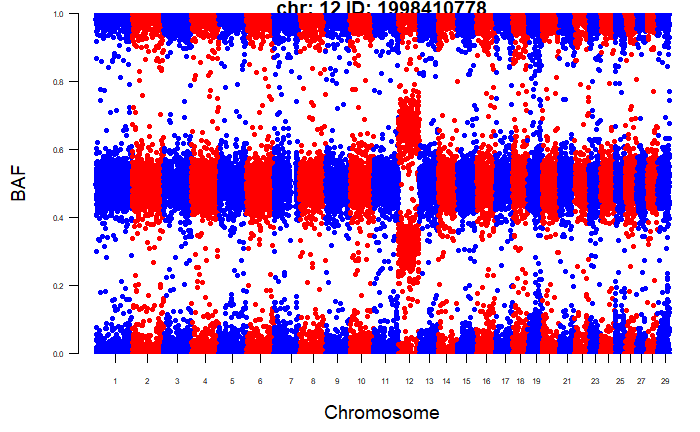C) | 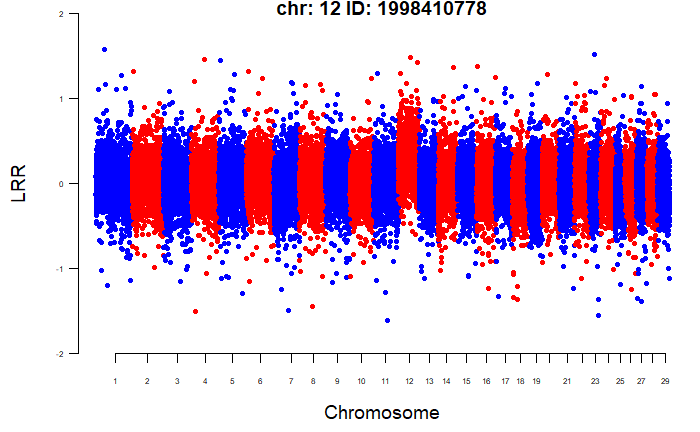 |
| 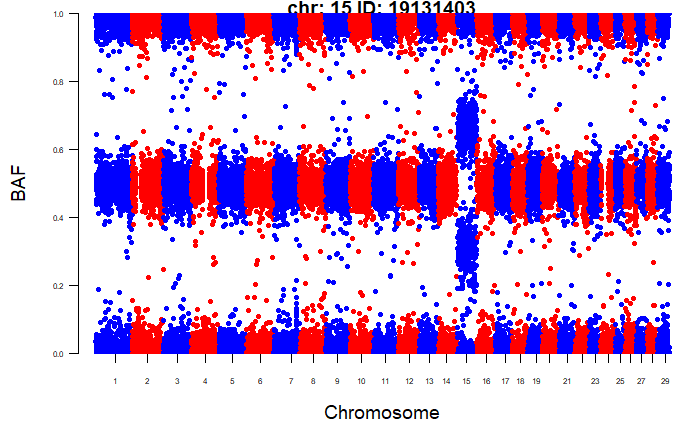D) | 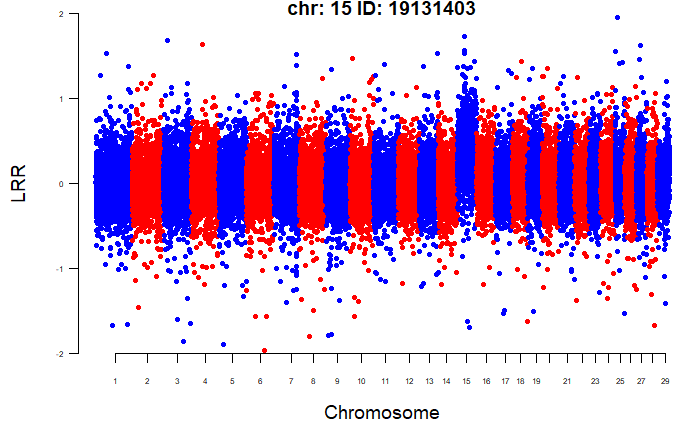 |
| 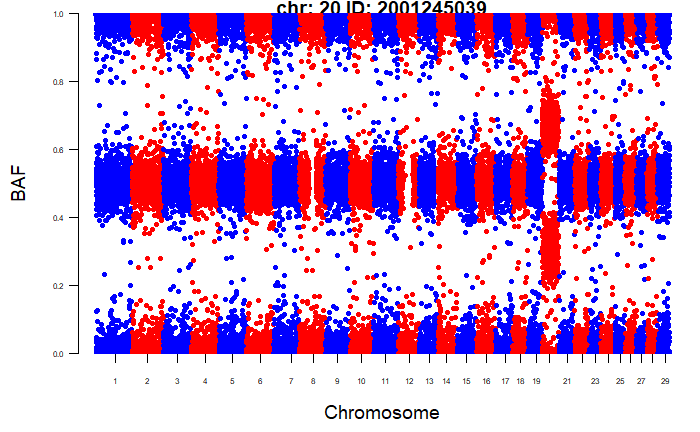E) | 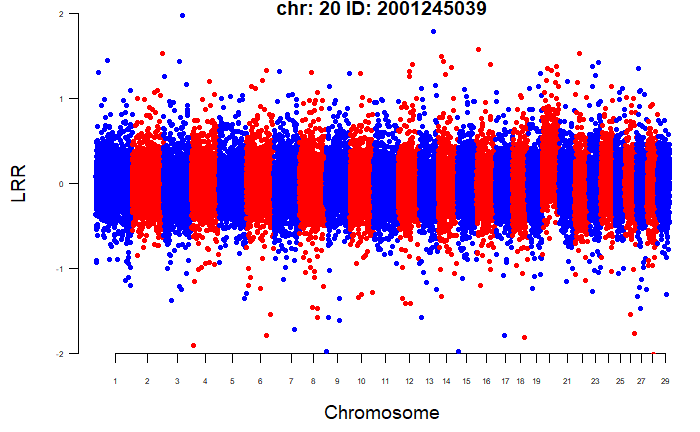 |
| 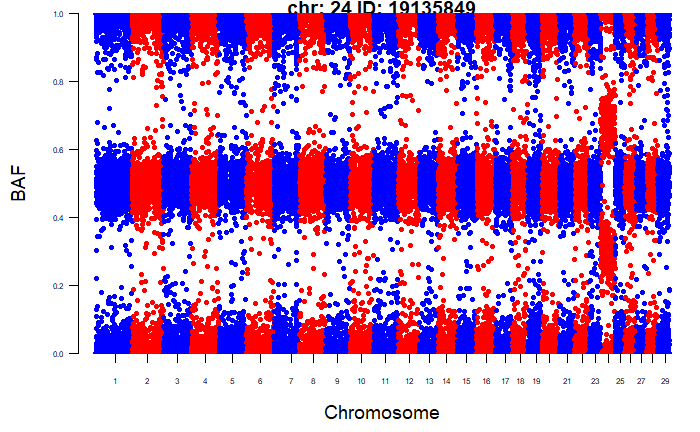F) | 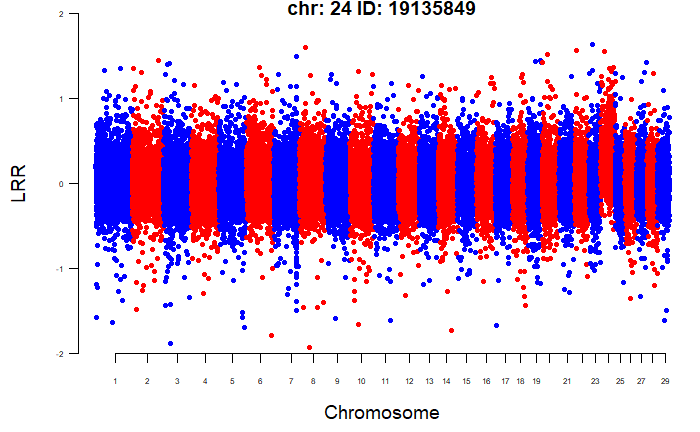 |
| 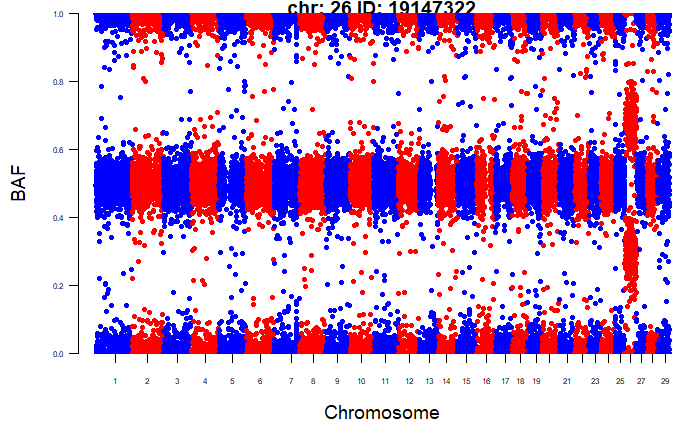G) | 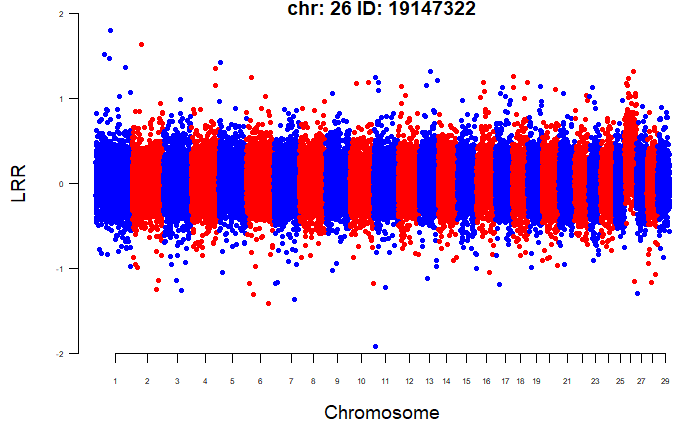 |
| 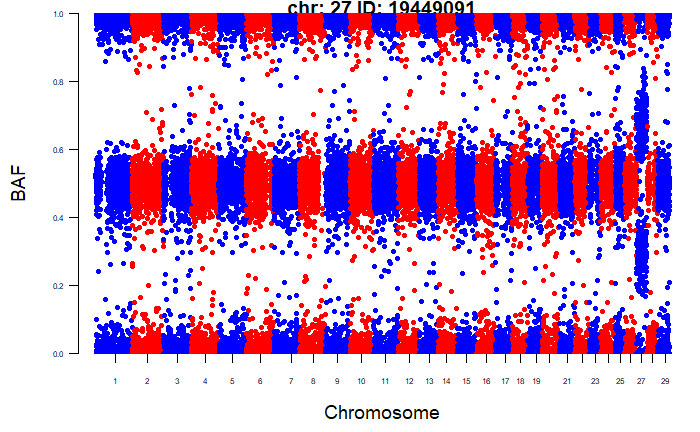H) | 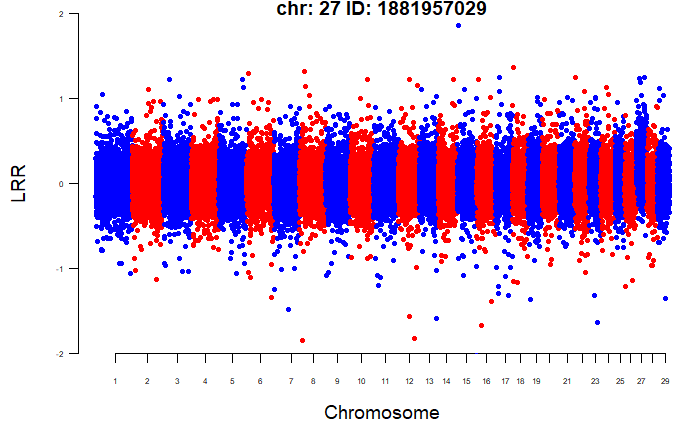 |
| 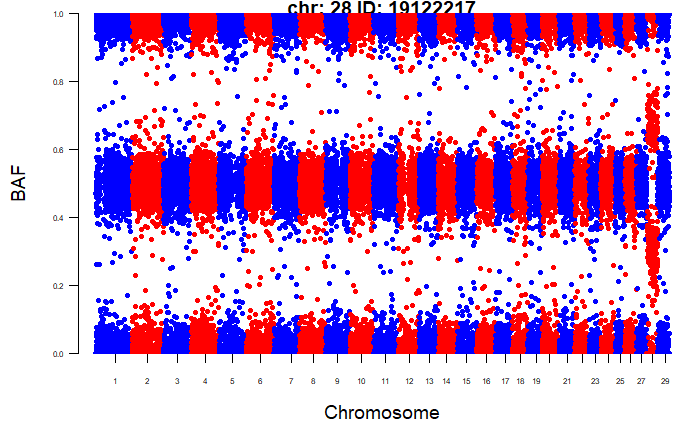I) | 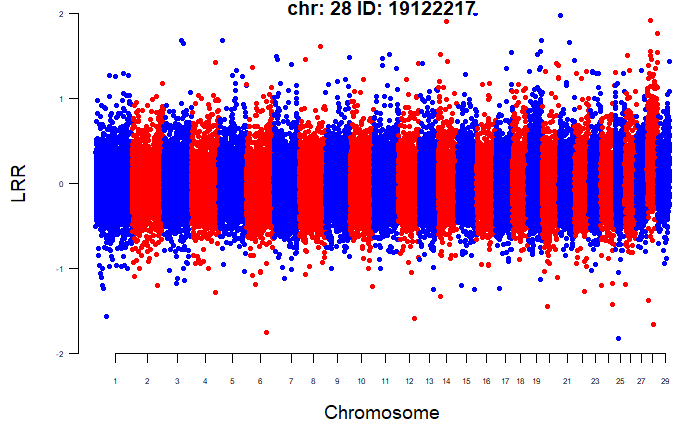 |
| 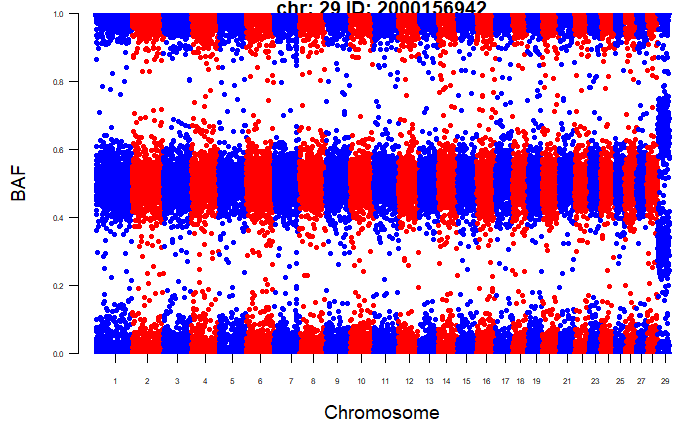J) | 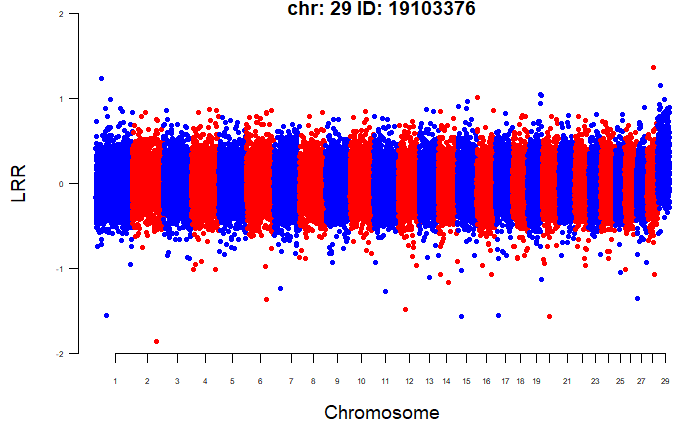 |

**Supplementary Figure 1.** Real life examples of Log R Ratio (LRR) and B-allele frequency (BAF) plots for each detected trisomy karyotype. **A)** trisomy on chromosome 4. **B)** trisomy on chromosome 6. **C)** trisomy on chromosome 12. **D)** trisomy on chromosome 15. **E)** trisomy on chromosome 20. **F)** trisomy on chromosome 24. **G)** trisomy on chromosome 26. **H)** trisomy on chromosome 27. **I)** trisomy on chromosome 28. **J)** trisomy on chromosome 29.
